# Guar gum/poly ethylene glycol/graphene oxide environmentally friendly hybrid hydrogels for controlled release of boron micronutrient

**DOI:** 10.1098/rsos.231157

**Published:** 2023-12-13

**Authors:** Muhammad Khalid Azeem, Atif Islam, Rafi Ullah Khan, Atta Rasool, Muhammad Anees Ur Rehman Qureshi, Muhammad Rizwan, Raa Khimi Shuib, Abdul Rehman, Ayesha Sadiqa

**Affiliations:** ^1^ Institute of Polymer and Textile Engineering, University of the Punjab, Lahore, Punjab, Pakistan; ^2^ Institute of Chemical Engineering and Technology, University of the Punjab, Lahore, Punjab, Pakistan; ^3^ School of Chemistry, University of the Punjab, Lahore, Punjab, Pakistan; ^4^ Department of Chemistry, Allama Iqbal Open University, Islamabad 47670, Pakistan; ^5^ Department of Chemistry, University of Lahore 54000, Pakistan; ^6^ School of Materials and Mineral Resources Engineering, Universiti Sains Malaysia, 14300, Penang, Malaysia; ^7^ Department of Polymer Engineering, National Textile University, Karachi campus, 74900, Karachi, Pakistan

**Keywords:** guar gum, poly (ethylene glycol), graphene oxide, hydrogels, boron release, sustained agriculture

## Abstract

The present study was aimed at synthesis of polymeric hydrogels for controlled boron (B) release, as B deficiency is a major factor that decreases crops yield. Thus, graphene oxide incorporated guar gum and poly (ethylene glycol) hydrogels were prepared using the Solution Casting method for boron release. 3-Glycidyloxypropyl trimethoxysilane (GLYMOL) was used as a cross-linker. Characterizations of hydrogels were carried out by Fourier Transform Infrared Spectroscopy (FTIR), Thermo-Gravimetric Analysis and Scanning Electron scope. The FTIR outcomes confirmed the existence of functional groups, bindings and development of hydrogel frameworks from incorporated components. The quantity of GLYMOL directly increased the thermal stability and water retention but decreased the swelling %. The maximum swelling for the hydrogel formulations was observed at pH 7. The addition of GLYMOL changed the diffusion from quasi-Fickcian to non-Fickcian diffusion. The maximum swelling quantities of 3822% and 3342% were exhibited by GPP (control) and GPP-8 in distilled water, respectively. Boron release was determined in distilled water and sandy soil by azomethine-H test using UV-Visible spectrophotometer while 85.11% and 73.65% boron was released from BGPP-16, respectively. In short, water retentive, water holding capacities, swelling performances, biodegradability and swelling/deswelling features would offer an ideal platform for boron release in sustained agricultural applications.

## 1. Introduction

Hydrogels are water absorbing polymeric networks that can act as water reservoir, soil conditioner and superabsorbent nutrient carrier that release fertilizers/chemicals in a sustained manner from their three-dimensional frameworks [1]. These also reduce leaching, sustain plants in drought and augment the physical properties of soil [2]. Today, agriculture is facing serious problems such as water stress, temperature, acidity, nutrient loss, excessive utilization of chemicals and salinity which is derived from urbanization, higher nutrient demands, overpopulation, urbanization, climate changes, global warming and land deficit [3]. Therefore, the researchers are interested in developing the methodological approaches to improve the crop yield by managing optimum supply of water, minerals and nutrients for the crops. The hydrogel technology is believed to be the ultimate and ideal approach to cope with the aforesaid challenges. In addition, the hydrogels possess pendant charge groups that govern pH responsive swelling behaviour dependent on the ionic charges, ionizable functional groups, p*K*a, ionic charges and concentration of polymer [4]. The swelling ability is owing to the distinctive characteristics of the hydrogels in different media such as water, pH, temperature and the ionic solutions, which permitted their usage in numerous fields like industry, biology, biomedicine and agriculture [5–7]. This swelling involves water diffusion inside the hydrogel that relaxes polymeric chains followed by swelling. Further, their superabsorbent nature promotes efficient obtainability, holding aptitude and irrigation capacities for plants [8].

Hydrogel platforms offer controlled and sustained release of fertilizer, pesticides, nutrients and herbicides [9]. The consumption of fertilizers had been increased tremendously by 5% per year since 1950 to 1980 [10]. The efficacy and affectivity of a fertilizer is dependent upon accessibility and matching requirements of the plants. Thus, the sustained release of fertilizer is an ideal strategy that not only enriches nutrients availability but also shrinks soil deprivation [11]. Outstanding non-hazardous, economical, photo stable, biodegradable, biocompatible and the environmentally friendlier nature of guar gum (GG) cellulose, chitosan, pectin, starch, Arabic gum and alginate derived hydrogels are in the favour of agriculture [12–16].

GG is a hydrophilic whitish yellow-coloured naturally occurring high molecular weight polysaccharide obtained from *Cyamopsis psoraloids*, a member of the Leguminosae family mainly cultivated in the regions of India and Pakistan. It has a straight chain of D-mannose units joined by β-(1→4)-glycosidic linkage and D-galactose units joined by (1→6)-linkage at alternate positions. The endospermic coat of guar seed pre-dominates the existence of mannose and galactose units, which are combined and called galactomannan components and an economical source of galactomannan that has a distinguishing position among biopolymers owing to its accessibility, modifiability, biodegradability, biocompatibility and non-toxic nature [17]. The capability of GG to make hydrogel is a substantial development, as it contains an –OH group that takes part in hydrogen bonding and ultimately plays a fundamental role in gel formation, swelling, solubility, viscosity, hydration, disintegration and biodegradation. Consequently, it is used as a gelling, stabilizing, binding, suspending, disintegrating and emulsifying agent [18].

Improvement in physico-chemical properties, enhancement in water retentive abilities of soil, film forming and nutrients carrying features of GG were explored for sustained release fertilizers such as di-ammonium phosphate, boron and zinc sulfate, etc. [19–21]. Likewise, we have also reported the sustained release of ammonium phosphate in our previous study [22]. The hydrogel films produced by GG are poor in mechanical properties; therefore, GG is blended with synthetic polymers [23].

Poly ethylene glycol (PEG) is a synthetic, hydrophilic and biocompatible polymer [24–26]. In addition, it is an FDA approved non-hazardous and non-immunogenic chemical explored for various biomedical, biological, commercial and industrial applications [27–29]. For hydrogel preparation, terminal hydroxyl functionalities present in PEG enable its modifiability for cross-linking. Thus, it modifies hydrogel architecture and improves their tensile properties [30]. It is also reported for tuning hydrophobicity and managing hydrophilic/hydrophobic balance [31]. GG has displayed compatibility with PEG. For instance, Songara *et al*. reported excellent moisture retention and soil conditioning capabilities of GG, PEG and methyl methacrylate hydrogel to cope with the water stress in sugarcane crops [32,33]. Therefore, in light of the aforementioned reflections, GG was blended with PEG for hydrogel preparation.

Graphene and its derivatives not only breed stabilities and mechanical strength, but also boost loading/release capacities in hydrogels [34]. Graphene oxide (GO) incorporated in hydrogel frameworks not only improves the mechanical properties, but also increases stabilities and surface area for loading and release of nutrients, drugs and fertilizers etc. [35,36]. Boron is an important micronutrient necessary for crop growth, quality and yield. It plays a critical role in cell division, metabolism, fertilization, germination and opening of stomata. However, its shortage causes leaf fall, undersized fruits, and low yields of cereals, beans, pulses and oil seeds. B deficiency has been reported for 132 crops for 60 years [37]. In acidic, high rainfall areas, the boron is the most deficient micronutrient for plants in soils due to leeching of clay minerals [38]. Hence, it is imperative to develop administrational strategies such as hydrogels to manage boron availability for plants in soil.

Hence, in the present study, we have made novel pH responsive GG/PEG/GO hydrogels using 3-glycidyloxypropyl trimethoxysilane (GLYMOL) for the sustained boron release. The hydrogels were characterized by fourier transform infrared spectroscopy (FTIR), thermo-gravimetric analysis (TGA) and scanning electron microscope (SEM). In addition, the swelling analysis was studied in distilled water (DW), non-buffers, buffers and ionic solutions (NaCl and CaCl_2_). Further, antimicrobial activity and *in-vitro* biodegradation were also studied. The boron release was studied in distilled water and sandy soil.

## 2. Material and methods

### 2.1. Chemical and reagents

Guar gum (Mw: 110 kDa) extra pure food grade was attained from Dabur India Limited. Poly (ethylene glycol) (Mw: 400), potassium dihydrogen phosphate (Mw: 141.96 g mol^−1^), GLYMOL (Mw: 236.34 g mol^−1^, 98%), acetic acid (Mw: 60.5 g mol^−1^, 99.8%,) GO powder (product no. 746034, Mw: 4239.48 g mol^−1^) and H_3_BO_3_ (Mw: 61.83 g mol^−1^) were acquired from Sigma Aldrich. Hydrochloric acid (Mw: 36.46, AnalaR) and sodium hydroxide (Mw: 40 g mol^−1^, Daejung) were used to prepare non-buffer solutions. In addition, disodium hydrogen phosphate (Mw: 177.99 g mol^−1^, Scharlau), sodium chloride (Mw: 58.5 g mol^−1^, Merck,), calcium chloride (Mw: 110.98 g mol^−1^ Merck, anhydrous) and potassium chloride (Mw: 74.55 g mol^−1^, Daejung) were brought into use for the preparation of phosphate buffer saline (PBS) solution.

### 2.2. Hydrogels formulation

A solution casting technique was used to synthesize GG/PEG/GO hydrogels by changing the quantity of GLYMOL. First of all, the separate solutions of 0.5 g of guar gum and 0.354 ml of PEG were prepared in distilled water (DW). Further, both solutions were mixed and stirred for 2 h at 60°C. GO (0.01 g) was taken in 10 ml of DW, sonicated for 30 min and added to the above mentioned blending mixture and agitated for an additional 1 h. Furthermore, the variable quantity of the silane cross-linker GLYMOL (80, 160, 240 and 320 µl) was dissolved in 5 ml of methanol separately and added to the aforesaid mixture and agitated for 1 additional hour. Finally, the solutions were poured into Petri dishes and dried to obtain the hydrogels coded as GPP (control), GPP-8, GPP-16, GPP-24 and GPP-32 incorporated with 80, 160, 240 and 320 µl of GLYMOL, respectively.

### 2.3. FTIR

FTIR explores important bonding interactions and the existence of functional groups involved in development interfaces among different constituents of the hydrogels, which were confirmed in scan range of 4000–400 cm^−1^ using thermo-scientific Nicolet iS10.

### 2.4. TGA analysis

Thermo-gravimetric analysis evaluates thermal properties and thermal stabilities of the fabricated hydrogels. A TGA Q50 instrument manufactured by USA Thermal Analysis (TA) was used for TGA analysis. 5 mg of each hydrogel specimen was placed in a pan that was put under nitrogen environment at the flow rate of 30 ml min^−1^. Temperature was raised with a factor of 10°C min^−1^ from 30 to 700°C.

### 2.5. Morphological study by SEM

SEM MIRA3 TESCAN linked with EDX at the Institute of Space and Technology, Islamabad, Pakistan was used to obtain SEM micrographs of simple hydrogels and KNO_3_ loaded hydrogels. Hydrogel samples were attached to carbon conductive tape for tungsten coating in sputter coater (Safematic CCU-010). The rate of coating was 0.8 nm s^−1^. After that, the inspection of the samples was accomplished in SEM at different magnifications.

### 2.6. Biodegradation in soil

Biodegradation rates of GG/PEG/GO hydrogels were evaluated monitoring weight loss in soil. 0.1 g of each hydrogel was placed in a perforated plastic bag and submerged in soil surface at the depth of 5 cm. These bags were removed, washed and dried every 2 days and weight loss was computed using equation (2.1) [39]. Whereas, *W*_o_ and *W*_1_ are the weights of hydrogels before and after biodegradation, correspondingly.

2.1 $$\mathrm{Biodegradation}\;(\text{\%})=\frac{W_{1}-W_{o}}{\;W_{o}}\;\times 100.$$

### 2.7. Effect of GG/PEG/GO on soil water holding and water retention capability

The stated hydrogels were also investigated for their impact on water holding capabilities of soil. For this purpose, 20 g of dry soil was considered as control (A). Similarly, 20 g of dry soil was mixed with 1% of GPP (control), GPP-8, GPP-16, GPP-24 and GPP-32 and sealed in 200-mesh nylon fabric was then weighted (*W*_o_) and coded as B, C, D, E and F, respectively. All the samples were soaked in up to saturation and weighted again (*W*_1_). Equation (2.2) was used to compute water holding capacity [39].

2.2 $$\mathrm{Water}\;\mathrm{holding}\;\mathrm{capacity}\;(\text{\%})=\frac{W_{1}-W_{o}}{\;W_{o}}\times 100.$$

On other hand, after 2 days, the soil samples were weighted every 24 h (*W*_i_) up to 20 days to determine water retention (%) of the soil by using equation (2.3)

2.3 $$\mathrm{Water}\;\mathrm{retention}\;(\text{\%})=\frac{W_{i}-W_{o}}{W_{i}-\;W_{o}}\times 100.$$

### 2.8. Swelling/deswelling studies

The following method was used to evaluate the swelling capabilities of the fabricated hydrogels. Pre-weighted quantity (10 mg) of the dried hydrogel specimen was placed in a suitable solvent. The excess solvent was removed with tissue papers after consistent intervals of time to govern the weight of the swollen hydrogel. The process was carried out until the establishment of swelling equilibrium in the same solvent. Equation (2.4) was used to compute swelling %.

2.4 $$\mathrm{Swelling}\;(\text{\%})=\frac{W_{s}-W_{d}}{W_{d}}\;\times 100\;.$$

Where, *W*_s_ and *W*_d_ represent swollen and dried weights of the hydrogels.

Later on, the swollen hydrogels were kept in an oven at 100°C until moisture was evaporated and the hydrogels reached their original dry weight. Subsequently, the dried hydrogels were again subjected to the above-mentioned swelling analysis.

### 2.9. Fertilizer loading and release

H_3_BO_3_, a water soluble compound of boron, was used as a source for the boron nutrient that was loaded on GPP-16. Briefly, 150 mg of H_3_BO_3_ was dissolved in 30 ml of DW. After that, it was added into GG/PEG/GO mixture followed by the addition of GLYMOL cross-linker (160 ul). The boron loaded hydrogels were prepared by the reaction of GG, PEG and GO and cross-linking was achieved by the GLYMOL followed by stirring for 4 h at 70°C. The content obtained was poured on a plastic Petri dish and allowed to settle and was then dried in an oven at 70°C and denoted as BGPP-16.

For the investigation of boron release, BGPP-16 was placed in 500 ml of DW at 25°C. A 3 ml sample was taken after 1, 3, 6, 9, 12, 18, 24, 36, 48, 60, 72, 96, 120, 144 and 168 h then treated with azomethine-H reagent [40]. As a result, the solution turned a dark yellowish colour. The intensity of the colour depends upon boron content that was calculated using a UV-Visible spectrophotometer at 420 nm [20]. Similarly, the pot culture technique was used to study the release of boron in the soil. For this purpose, sandy soil was acquired from Pakistan Water and Research Council (PWRC) farm and an azomethine-H test was used for the evaluation of boron content, which was 0.22 mg kg^−1^. A total of 200 g of air dried soil was placed in plastic pots. In addition, 0.5 g of BGPP-16 (containing 75 mg of boron) was enclosed in a permeable bag made of chiffon fabric and added into the centre of the soil in the pot. The pots were placed at 25–30°C temperature. Throughout the experiment, the soil was maintained at 1/3 for water by weighing and adding DW if required [41]. After 2, 4, 6, 8, 10, 12, 14, 16,18, 20, 25 and 30 days, the buried bags were removed from their corresponding beakers, their soil was dried and mixed in a uniform manner followed by taking 10 g of soil sample for the boron analysis using the standard protocol method [42].

### 2.10. Statistical analysis

The statistical analysis of the data was carried out using Origin Pro 8.51 software made by Origin Lab Corporation (Northampton, USA). The statistical variances were computed by one-way ANOVA and Tukey tests. The *p* < 0.05 was considered significant and data was provided as an average of ± standard deviation.

## 3. Results and discussion

**Figure 1.**
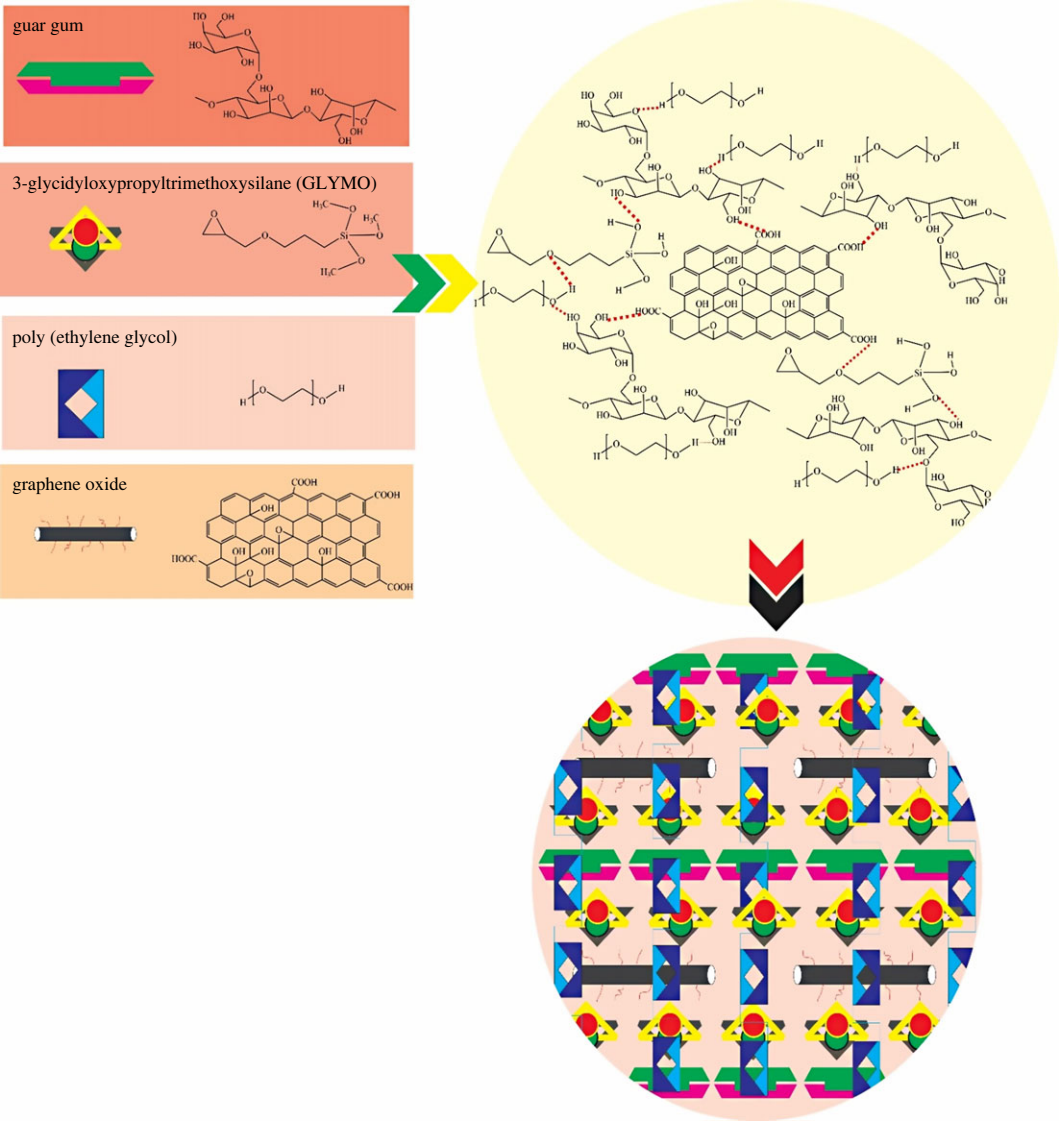
The proposed scheme for GG/PVA/GO hydrogel cross-linked by GLYMOL.

The scheme suggested for the fabricated hydrogel for the boron release is represented in figure 1.

### 3.1. FTIR analysis

**Figure 2.**
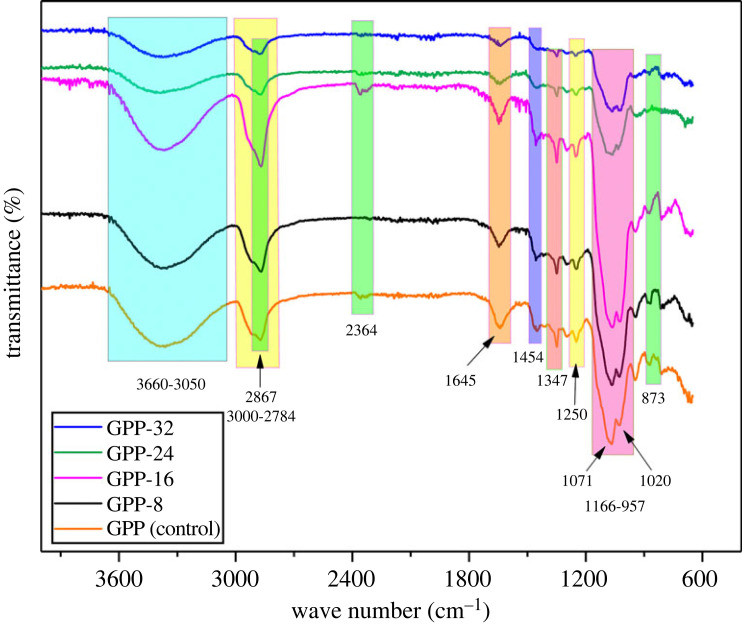
FTIR spectrum of the prepared hydrogels.

FTIR spectra for GPP (control), GPP-8, GPP-16, GPP-24 and GPP-32 are depicted in figure 2 which explores the important interfaces in the hydrogels. The hydrogels comprised GG, PEG, GO and GLYMOL. The broad stretching band appearing at 3050–3660 cm^−1^ is due to –OH^−^ groups involved in the hydrogen bonding, which is present in GG, PEG and GO. GG inherit numerous –OH^−^ confirmed by its molecular structure depicted peak in the 3050–3660 cm^−1^ region [43]. In addition, the peak observed at 2867 cm^−1^ represents the polymer linked –OH^−^ stretching in the 2784–3000 cm^−1^ region. The peak detected at 1347 cm^−1^ is accredited to –OH^−^ bending vibrations [44].

The fundamental interaction is siloxane linkage (Si-O-Si) between GG and GLYMOL that is confirmed by the peak at 1020 cm^−1^. Figure 2 also demonstrates the representative peaks related to PEG that are indicated by the peaks at wavenumbers 3600, 2900, 1070 and 957 cm^−1^ [45]. The peak at 3600 cm^−1^ is due to the –OH stretching being overlaid in the 3050–3600 cm^−1^ region. In addition, 2900 cm^−1^ is the peak for –C-H stretching in the –CH_2_ group [46]. The peaks at 1454, 1070, 957 and 800 cm^−1^ are assigned to C-O stretching, hydroxyl bending and C-O-C symmetrical stretching, respectively [47]. The wide peak 3050–3600 cm^−1^ endorses the –OH group, whereas the peak at 1645 cm^−1^ explains the symmetrical deformation mode of COO^−^ present in GO. Likewise, the epoxy (C-O-C) stretching vibrations were evident at 1155 cm^−1^ and 1043 cm^−1^ observed in the 957–1166 cm^−1^ region [48]. The characteristic peak for –C = O involved in the hydrogen bonding is absent at 1749 cm^−1^, which establishes the existence of the hydrogen bonding in the GG/PEG/GO hydrogel blends [4]. Figure 2 endorses the presence of all the functional groups associated with the hydrogel components.

### 3.2. Thermal analysis

**Figure 3.**
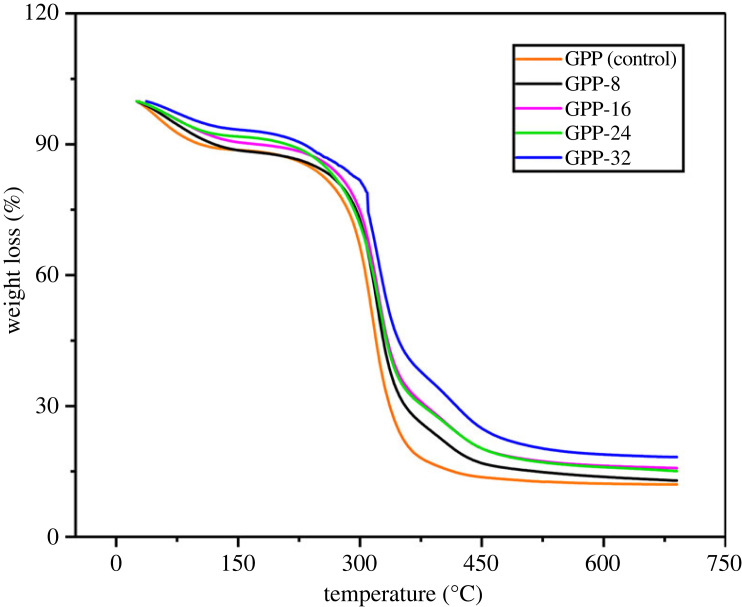
Thermo-grams of GG/PEG/GO hydrogels.

TGA investigations of GPP (control), GPP-8, GPP-16, GPP-24 and GPP-32 have been displayed in figure 3. Thermal stability of the hydrogels depicted a direct relation to the quantity of GLYMOL cross-linker. Thus, GPP-32 presented higher stabilities as it comprised 320 µl of GLYMOL while GPP (control) is least stable as it lacks GLYMOL. Similarly, the increase in thermal resilience was observed in GPP-8, GPP-16, GPP-24 and GPP-32 due to the presence of 80, 160, 240 and 320 µl of GLYMOL, respectively. Cross-linkers directed stability inherited in its five cross-linking points that are involved in the hydrogen bonding and the covalent interactions, thus the hydrogel structure becomes compact. This is how the rate of biodegradability, thermal stability, compactness and mechanical properties of hydrogels are improved by cross-linker. GG/PEG/GO fabricated hydrogels established decay in three stages. The first stage comprised weight loss that ranges from 30–250°C which is attributed to the water and moisture elimination. The second phase ranged from 250–350°C where degradation in GG was initiated and weight loss occurred due to the elimination of side chains. The final phase belongs to the decomposition from 350–700°C credited to the onset degradation of GG backbone [49].

### 3.3. SEM analysis

**Figure 4.**
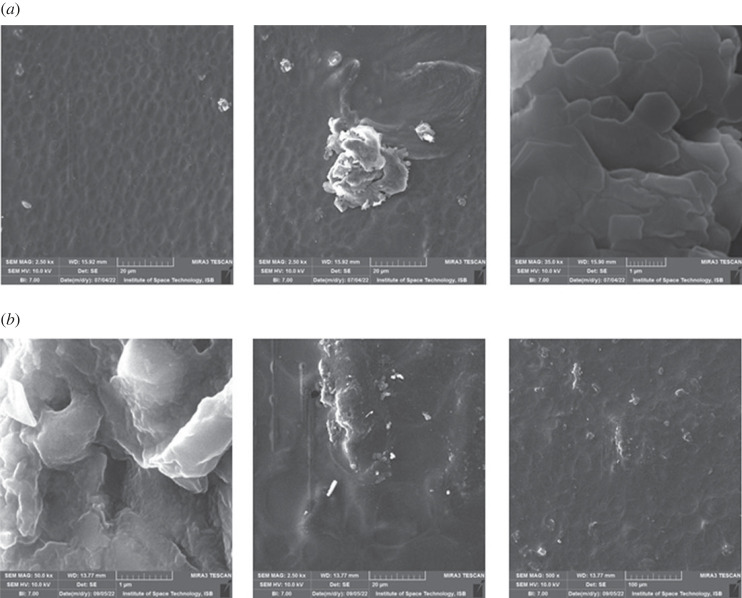
(*a*) SEM of GPP-16 at various magnifications. (*b*). SEM micrographs of BGPP-16 (H_3_BO_3_ loaded GPP-16).

SEM micrographs of GPP-16 hydrogels are portrayed as non-uniform, coarse and heterogeneous surfaces. At higher magnifications multifaceted and complex structural sites are also visible on the hydrogels surfaces as a result of GLYMOL crosslinking GG, PEG and GO depicted in figure 4*a*. These coarse surfaces play an essential role in holding and retaining fertilizers [28]. On the other hand, figure 4*b* showed H_3_BO_3_ loaded GGP-16, which offered the similar heterogeneous spots and rough surfaces along with H_3_BO_3_ molecules. It is evident that the topography of BGPP-16 endorsed effective synthesis and H_3_BO_3_ loading in GLYMOL cross-linked GG/PEG/GO hydrogel.

### 3.4. Biodegradation test

**Figure 5.**
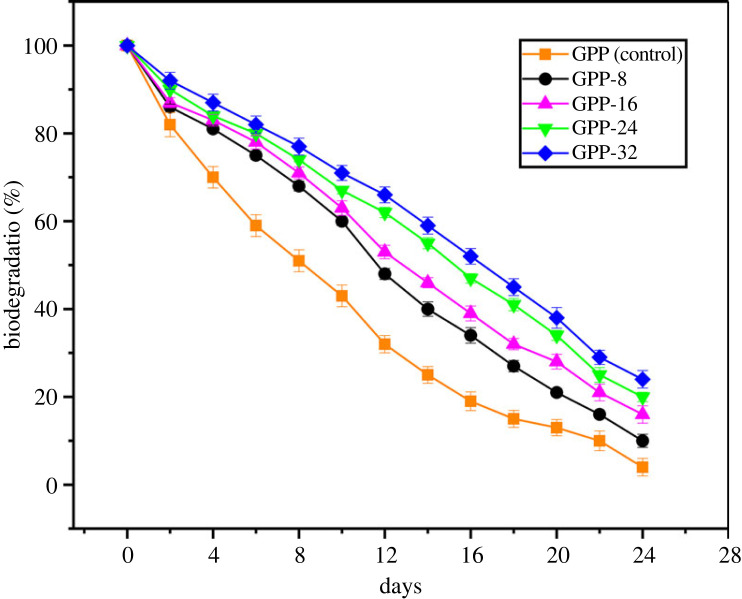
Biodegradation % of GPP (control), GPP-8, GPP-16, GPP-24 and GPP-32 in soil.

The biodegradation ability of the prepared hydrogels is ascribed to the presence of GG, which is comprised of simple sugars, galactose and mannose in 1 : 1 : 6 ratio [50]. The biodegradation of each hydrogel specimen was examined in the soil illustrated in figure 5. Resultantly, GPP (control), GPP-8, GPP-16, GPP-24 and GPP-32 demonstrated 93%, 90%, 86%, 83% and 80% biodegradation, respectively. In addition, the biodegradation in the hydrogels is inversely related to the quantity of GLYMOL. This behaviour can be explained as the GLYMOL comprising five cross-linking points that played a part in the strong hydrogen and covalent bonds and strong hydrogen bonding [51].

### 3.5. Water holding capacities

**Figure 6.**
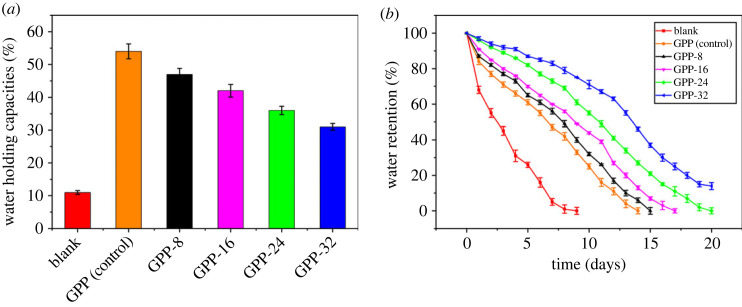
(*a*) Water holding capacities and (*b*) water retention of fabricated hydrogels.

The computed water holding capacities of GPP (control), GPP-8, GPP-16, GPP-24, GPP-32 and blank were 54%, 47%, 42%, 36%, 31% and 11% as shown in figure 6*a*. The water holding capacities of the soil have increased by the addition of 1% of hydrogel formulations in 20 g of soil. However, the maximum water holding ability was depicted by the GPP (control). It is evident that the addition of the claimed hydrogel formulations modified the water holding capacity of the soil which was attributed to their hydrophilic nature. Furthermore, higher surface area, depicted in the SEM micrograph, also endorsed the infiltration of the water molecules in voids inside the hydrogels.

### 3.6. Water retention

Figure 6*b* represented the water retention capabilities of the designed GG/PEG/GO hydrogels by varying the quantity of GLYMOL.

It is recognizable that blanks have lost all irrigated water in 5 days. However, the water retentive abilities of the 1% hydrogel mixed soil decreased after 16 days. In addition, the maximum water holding capacities were showcased by the GPP-32. This might be due to the hydrophilic groups and the nature of the biopolymeric guar gum based hydrogels that can entrap more water due to the hydrogen bonding, the porosity and the higher surface area [52].

### 3.7. Swelling in DW

**Figure 7.**
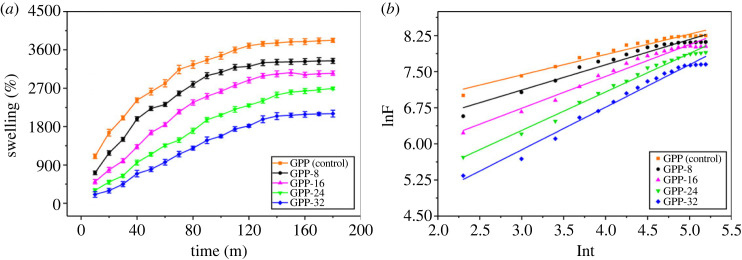
(*a*) Swelling trend of the synthesized hydrogels in DW, (*b*) calibration graph for computing the diffusion parameters.

Hydrogels engross water that is described by their hydrophilic nature. The examined swelling results of GG/PEG/GO hydrogels are provided in figure 7*a*.

It is visible that GPP (control) exhibited the highest swelling 3822% among prepared hydrogels while the least swelling % was shown by GPP-32. Therefore, the addition of the cross-linker (GLYMOL) inversely affected the swelling % because it contained five cross-linking points that took part in the stronger hydrogen and the covalent bonds, which decreased the swelling %. Similarly, GPP-8 has shown higher swelling 3342% than GPP-16, GPP-24 and GPP-32 and lower than GPP (control). In addition, every hydrogel formulation represented a linear rise in swelling with respect to time. This distinctive action and swelling aptitude makes these hydrogels an appropriate vehicle for the agricultural applications. The higher quantity of GLYMOL improved stability by stimulating binding among the hydrogel constituents which henceforth lessened the swelling rate. The entry of water molecules into the hydrogel framework is produced by the swelling and the diffusion phenomenon. The mechanism of swelling and the diffusion model could be explained by equation (3.1). While F is fractional swelling, *k* is rate constant for swelling, *t* is time taken to swell and *n* is swelling exponent. The values of ‘*k*’ and ‘*n*’ were calculated from the calibration curves described in figure 7*b* and data is given in table 1.

3.1 $$F=kt^{n}.$$

**Table 1.** Diffusion parameters GLYMOL cross-linked GG/PEG/GO hydrogels. SE is the standard error in slope.

parameters	hydrogel samples				
	GPP (control)	GPP-8	GPP-16	GPP-24	GPP-32
adj. *R*^2^	0.96089	0.94957	0.97198	0.98738	0.98256
*R*^2^ (COD)	0.9632	0.9525	0.9736	0.9881	0.9836
slope (*n*)	0.4229	0.5245	0.6607	0.799	0.8837
SE	0.02067	0.02927	0.02719	0.0219	0.02854
intercept	6.1638	5.5444	4.7571	3.8822	3.226
*k*	475.231	255.801	116.408	48.5309	25.1787

It is understandable from the data obtained from the calibration curves that solvent molecules diffused into hydrogel matrix in GPP (control) Quasi-Fickian stereoselective diffusion model as the value of *n* < 0.5. However, the cross-linking produced by the addition of GLYMOL transformed the entry of the water molecules inside the hydrogel matrices via non-Fickian diffusion in GPP-8, GPP-16, GPP-24 and GPP-32 as a value of *n* > 0.5 [22]. The value of ‘n’ is less than 1 which indicates the diffusion process involved in release and transport of the solvent molecules in all the hydrogel samples [53].

### 3.8. Swelling in ionic solutions

**Figure 8.**
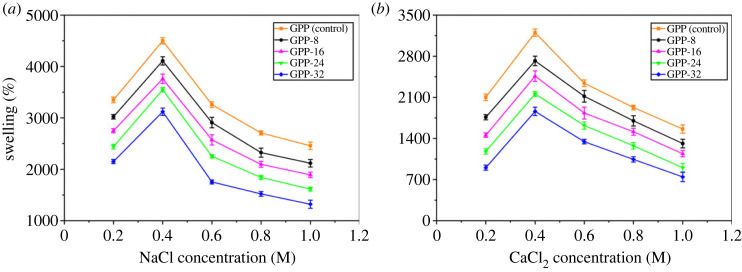
The swelling pattern of GGP (control), GPP-8, GPP-16, GPP-24 and GPP-32 in ionic solutions.

Figure 8*a,b* demonstrates the swelling behaviour of the fabricated hydrogels in NaCl and CaCl_2_ solution, respectively. Both electrolytic solutions possess the same anion but differ in cations; therefore, charge to size ratio is different, which has an impact on the swelling volumes. It is evident from the figures that the increasing concentration of the electrolytic solutions increased the ionic strength, which ultimately decreased the swelling %. In addition, more swelling has been observed in NaCl than in CaCl_2_ due to higher ionic charge.

### 3.9. Swelling/deswelling studies

**Figure 9.**
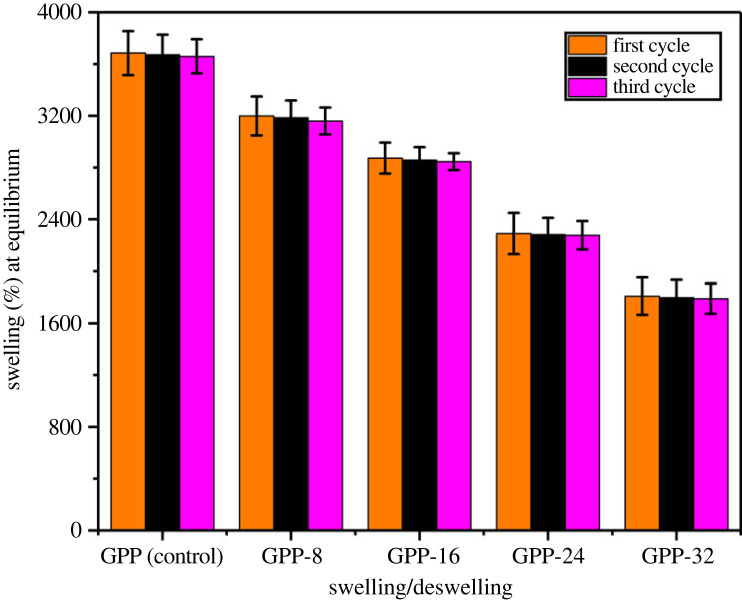
Swelling/deswelling cycles of the fabricated hydrogels.

The deswelling studies were also conducted for three swelling/deswelling cycles to investigate the reusability of our reported hydrogel. GG/PEG/GO blends depicted comparable swelling/deswelling upon successive cycles; hence, the hydrogels can absorb water and nutrients upon their availability, which are anchored by the plant roots under water and the nutrient stress conditions [54]. The results are demonstrated in figure 9. Our results also revealed that these hydrogels are reusable time and again. This significant potential is due to the presence of GO and GLYMOL cross-linker that sustain hydrogels for agronomic applications.

### 3.10. Boron release studies

**Figure 10.**
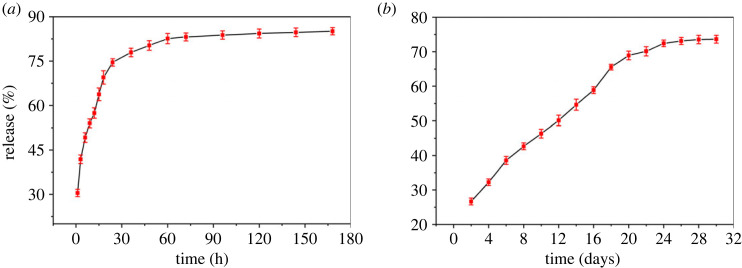
Boron controlled release from BGPP-16 (*a*) DW (*b*) sandy soil.

The GPP (control) and GPP-8 has good swelling and water holding capacities but lower water retention. On other hand, GPP-32 has the lowest swelling and water holding capacities and the highest water retentive capabilities. Therefore, GPP-16 is selected for loading/release study of B because of better swelling, water holding capacity and water retention. Figure 10*a* represented boron release in DW from BGPP-16. In the first phase, 41% of boron was released in three hours which might be attributed to the concentration gradient and the release of free boron from the surface of the hydrogel. 8% of boron was release in 3–6 h that is ascribed to the dissolution of H_3_BO_3_ inside the hydrogel creating a difference in osmotic pressure [55]. In the third phase, the hydrogel acquired swelling equilibrium and further boron release was attributed to the diffusion controlled release of boron from hydrogels. At this stage, the release is constant and independent of concentration [56]. In addition, the concentration gradient between the hydrogel and the surrounding medium for boron also decreases which slows down the release of boron.

Overall 85.11% f boron was determined in DW (pH 7). Similar studies were reported for the boron release in literature [57]. The sandy soil has lower water holding and water retention properties and as a result nutrients leeching takes place that affects plant growth. As fabricated GLYMOL cross-linked GG/PEG/GO hydrogels improved water retention and water holding capabilities of sandy soil, the boron release was studied in sandy soil (sand 79.9%, clay 8.2% and slit 11.9%) at pH 7.79. The release pattern of boron in sandy soil is demonstrated in figure 10*b*. Similar patterns for the boron release have been presented with 73.65% of the boron release in the soil (pH 7.79). However, a comparatively slower release was observed in the soil due to the lesser availability of water that not only limited the boron diffusion but also minimized the dissolution. A release % of boron from the fabricated GG/PEG/GO hydrogels displayed the encouraging results for their application in the boron deficient soils to improve crop yield and plant growth.

## 4. Conclusion

GG/PEG/GO biodegradable hydrogels were successfully prepared using the solution casting technique. The fabricated hydrogels were characterized by FTIR, TGA and SEM analysis. GPP (control) and Gpp-8 have revealed maximum swelling 3822% and 3342% respectively in DW. The swelling capabilities of the hydrogels depicted inverse relation to the amount of cross-linker (GLYMOL). The hydrogels displayed biodegradability for the agronomic applications. In addition, GG/PEG/GO not only displayed improvement in water holding capacities but also modified water retention. The swelling actions of the hydrogels encouraged their utilization for the controlled release of nutrients, fertilizer and pesticides. Further, the deswelling studies confirmed their reusability with time. In seven days (168 h), 85.11% of boron was released in DW. Moreover, 73.65% of boron was released in sandy soil up to 30 days. It is established that these hydrogels are capable of playing a substantial role in agronomic applications for the sustained release of fertilizers, pesticides and nutrients.

## Data Availability

The digital data were transformed into figures which are uploaded during submission of files and also represented in figures incorporated in the main manuscript file. The digital datasets, codes, results and supported data of present submission is uploaded on Dryad Digital Repository: https://doi.org/10.5061/dryad.crjdfn39v [58].
